# Lipid Metabolic Changes and Mitochondrial Stress in Ethanol-Treated Alveolar Type II Epithelial Cells: Initial Events Leading to Alcoholic Chronic Lung Disease

**DOI:** 10.3390/cells14221817

**Published:** 2025-11-19

**Authors:** Mukund Srinivasan, Bhupendra S. Kaphalia

**Affiliations:** Department of Pathology, The University of Texas Medical Branch, Galveston, TX 77550, USA; msprathi@utmb.edu

**Keywords:** Alcohol use disorder, alveolar type II epithelial cells, fatty acid ethyl esters, surfactants, oxidative stress, ER stress, mitochondrial stress

## Abstract

Alcohol use disorder (AUD) predisposes individuals to pneumonia, acute respiratory distress syndrome, and chronic obstructive pulmonary disease, yet the mechanisms underlying alcohol-related lung disease (ARLD) remain unclear. Alveolar type II (AT2) epithelial cells play a central role in ethanol (EtOH) metabolism, surfactant production, alveolar repair, and pulmonary innate immunity. To examine EtOH-mediated effects, immortalized human AT2 cells were treated with 22–130 mM EtOH for 6 h (concentration-dependent) and 65 mM EtOH for 6–72 h (time-dependent). Cytotoxicity, inflammation, surfactant lipid/protein dysregulation, fatty acid ethyl ester (FAEE) formation, cellular stress responses, AMP-activated protein kinase (AMPKα) signaling, and mitochondrial function were analyzed. EtOH disrupted surfactant homeostasis by reducing dipalmitoylphosphatidylcholine and surfactant protein C (SP-C). Importantly, EtOH inactivated AMPKα, downregulated CPT1A (involved in β-oxidation of fatty acids), and upregulated lipogenic proteins ACC1 and FAS, accompanied by increased ER stress markers (GRP78, p-eIF2α, and CHOP). Expression of carboxyl ester lipase (FAEE-synthesizing enzyme) and FAEE levels increased with EtOH exposure, further exacerbating oxidative and ER stress, impairing mitochondrial energetics, ATP production, and AT2 cell function. These findings suggest that EtOH-induced FAEE formation, dysregulation of AMPKα-CPT1A signaling, and surfactant contribute to AT2 cell dysfunction and play a critical role in the pathogenesis of ARLD.

## 1. Introduction

Alcohol use disorder (AUD) and abuse cost ~$250 billion to the U.S. economy and 100,000 deaths each year [1]. Although the hepatotoxic effects of alcohol are well-documented, increasing evidence suggests that AUD is also an independent risk factor for pulmonary complications, including acute respiratory distress syndrome (ARDS), pneumonia, pulmonary fibrosis, and chronic obstructive pulmonary disease (COPD) [2,3,4,5]. About 10% of the U.S. population diagnosed with AUD have a tenfold higher risk of developing pneumococcal pneumonia and a fourfold higher risk of pneumonia-related mortality compared with nondrinkers [6,7]. Similarly, an increased mortality rate has been reported among individuals with AUD diagnosed with ARDS, which is a fourth leading cause of death [2]. Chronic alcohol consumption/AUD impairs pulmonary immune defense, disrupts epithelial barrier integrity, and causes oxidative stress, thereby contributing to the pathogenesis of ARDS, pneumonia, COPD, and pulmonary fibrosis [3,4,8,9,10,11,12,13]. Alcohol-associated lung phenotype, commonly referred to as alcohol-related lung disease (ARLD), is clinically comparable in severity and mortality to alcohol associated liver disease [2,14,15]. However, it remains unclear whether chronic alcohol consumption/AUD alone is sufficient to cause persistent lung injury such as pulmonary fibrosis, or directly contributes to ARDS, pneumonia, and COPD. Despite strong clinical correlations, the precise cellular and molecular mechanisms by which chronic alcohol [ethanol (EtOH)] disrupts pulmonary alveolar epithelial function remain incompletely understood.

Approximately 90% of ingested alcohol is oxidatively metabolized to acetaldehyde in the liver by alcohol dehydrogenase 1 (ADH1), whose activity is significantly reduced in individuals with a history of AUD and in experimental animals fed chronic EtOH [16,17,18,19,20]. Such metabolic state of the liver substantially increases the body’s burden of alcohol, facilitating its circulation to extrahepatic organs, including the lungs. Under these metabolic conditions, alcohol undergoes non-oxidative metabolism to form fatty acid ethyl esters (FAEEs), catalyzed by FAEE synthase [21]. FAEEs persist and accumulate in body tissues and biological fluids, significantly disrupting cellular energetics, signaling pathways, and organelle functions [21,22,23,24].

Significantly high levels of FAEEs have been detected in the lungs of EtOH-fed animals pretreated with hepatic ADH inhibitor [25,26], as well as in hepatic ADH-deficient (ADH^-^) deer mice fed chronic EtOH [27]. These mice exhibited significant oxidative stress, endoplasmic reticulum (ER) stress, and inflammation in the lungs [27]. Moreover, we have demonstrated concentration-dependent formation of FAEEs in primary human alveolar macrophages (AMs) treated with EtOH [28]. Therefore, evaluating the cytotoxicity of FAEEs using various pulmonary cells is of great interest to understand the metabolic basis and mechanisms underlying ARLD.

The distal respiratory system exhibits extensive cellular heterogeneity, comprising approximately 40 distinct cell types [29,30]. About 95% of the alveolar surface area in the lungs is covered by large, thin squamous alveolar type 1 (AT1) epithelial cells, which facilitate gaseous exchange; the remaining 5% is covered by small, cuboidal alveolar type 2 (AT2) epithelial cells [31]. AT2 cells are the primary cells responsible for the synthesis and secretion of surfactant phospholipids such as dipalmitoylphosphatidylcholine (DPPC, a major pulmonary surfactant) and surfactant proteins (SP-A, SP-B, SP-C, and SP-D), which reduce alveolar surface tension and prevent lung collapse during respiration. They also serve as progenitors for AT1 cells and play a key role in epithelial repair and innate immunity [31,32]. Dysregulation of AT2 cell function has been implicated in various pulmonary diseases, including ARDS and idiopathic pulmonary fibrosis [32]. Therefore, understanding the cellular and molecular mechanisms of EtOH-induced toxicity in AT2 cells is therefore essential to delineate the etiopathogenesis of ARLD.

Emerging evidence suggests that alcohol disrupts cellular metabolism and redox homeostasis in the lungs [3,33]. EtOH and its metabolites induce oxidative stress, ER stress, inflammatory responses, impair phagocytic function, dysregulate cellular and mitochondrial bioenergetics, potentially leading to cellular injury in primary human AMs, bronchial epithelial cells, and airway smooth muscle cells [28,34,35]. However, the direct effect of EtOH on AT2 cell function, lipid metabolism, surfactant production, FAEE formation, and mitochondrial bioenergetics has not been well studied.

AMP-activated protein kinase α (AMPK), a master regulator of cellular energetics, mitochondrial function, and lipid metabolism, protects against oxidative/ER stress and inflammation [36,37,38]. Various pulmonary cells treated with EtOH at concentrations relevant to those reported in chronic alcoholic subjects, deactivate AMPKαleading to metabolic reprogramming and cellular dysfunction [28,34,35,36,37,38]. Therefore, given the central role of AMPKα activation in coordinating and maintaining various cellular functions and homeostasis, we believe that understanding the role of AMPKα in ARLD offers a potential therapeutic avenue to pursue.

Although chronic EtOH exposure has been linked to oxidative stress, inflammation, and immune dysfunction in the lungs, the metabolic and signaling mechanisms underlying alveolar epithelial injury remain poorly defined. The present study introduces a novel mechanistic concept centered on the FAEE–AMPKα axis, a previously unrecognized pathway contributing to the pathogenesis of ARLD.

We hypothesize that EtOH-induced formation and accumulation of FAEEs in AT2 cells impair alveolar epithelial function by suppressing AMPKα signaling, thereby promoting oxidative and ER stress, mitochondrial dysfunction, and surfactant dysregulation. Therefore, we assessed metabolism of EtOH, formation of FAEE, cytotoxicity, inflammation, oxidative and ER stress, mitochondrial bioenergetics, and surfactant phospholipid/protein expression in immortalized human AT2 cells treated with EtOH, to delineate the mechanistic contribution of the FAEE–AMPKα axis to ARLD and identify potential molecular targets for early therapeutic intervention.

## 2. Materials and Methods

### 2.1. Antibodies and Reagents

Primary antibodies against acetyl CoA carboxylase 1 (ACC1; 265 kDa; Cat # 4190) and phospho (p)-ACC1 (Ser 79; 280 kDa; Cat #3661); AMPKα (62 kDa; Cat # 5831) and p-AMPKα (Thr 172) (62 kDa; Cat # 2535); Ca^2+/^calmodulin-dependent protein kinase, kinase β (CaMKKβ; 60, 50 kDa; Cat# 4436); and p-CaMKKβ (Thr286) (60, 50 kDa; Cat# 12716); liver kinase B 1 (LKB1; 54 kDa; Cat# 3050) and p-LKB1 (Ser 428) (54 kDa; Cat # 53482); fatty acid synthase (FAS; 273 kDa; Cat # 3189); and β-actin (45 kDa; Cat # 4970) were obtained from Cell Signaling Technology (Danvers, MA, USA).

Antibodies against carnitine palmitoyl transferase 1A (CPT1A; 88 kDa; Cat # 128568), cytochrome P450 2E1 (CYP2E1; 50 kDa; Cat# ab28146), and spliced (s)/unspliced (u) X-binding protein (XBP1; 40 kDa and 29 kDa; Cat # 37152) were purchased from Abcam Inc (Cambridge, MA, USA). Antibodies against inositol-requiring transmembrane kinase/endoribonuclease 1α (IRE1α; 110 kDa; Cat # NB100-2324), and p-IRE1α (Ser 724) (110 kDa; Cat # NB100-232) were obtained from Novus Biologicals (Littleton, CO, USA). Antibodies against carboxyl ester lipase (CEL; 74 kDa; Cat# sc-377130), CHOP (31 kDa; Cat #MA1-250), and protein kinase RNA-like endoplasmic reticulum kinase (PERK; 150 kDa; Cat # 100-401-962), were procured from Santa Cruz biotechnology, Inc. (Dallas, TX, USA), Thermo Fisher Scientific (Houston, TX, USA), and Rockland (Limerick, PA, USA), respectively.

All other chemicals and reagents were purchased from Sigma Aldrich (St Louis, MO, USA) or ThermoFisher Scientific (Houston, TX, USA).

### 2.2. Cell Culture Studies

Primary human alveolar type II epithelial (AT2) cells exhibit limited proliferation in vitro and therefore cannot be passaged and expanded for extensive studies. In addition, they undergo phenotypic drift and lose physiological characteristics and function as they multiply or grow under cultured conditions [39,40]. Therefore, immortalized human AT2 cells, which are phenotypically stable, easier to maintain, and offer several advantages such as extended lifespan and reduced variability between the experiments, were used in this concentration and time-dependent EtOH toxicity studies.

The immortalized Human Alveolar Type II Epithelial Cells -SV40T+hTERT obtained from Applied Biological Materials Inc. (Cat # T0206, Richmond, BC, Canada) were seeded (~5000 cells/cm^2^) into the cell culture flask and grown in PriGrow III medium (Cat # TM003, Applied Biological Materials Inc., Richmond, BC, Canada). The medium was supplemented with 10% fetal bovine serum (FBS), 25 mM HEPES, and 1% Penicillin/Streptomycin solution. Cultures were incubated under standard conditions as described previously [34,35].

A concentration and time-dependent study was conducted to determine alcohol-associated toxicity in the AT2 cells. Blood alcohol concentration (BAC) of 300 mg/100 mL (65 mM) or more is commonly reported in chronic alcoholic subjects [41]. A 0.08% of alcohol corresponds to legal limit recommended for the alcohol in the United States [42]. Therefore, 22 (near legal limit), 65, and 130 mM EtOH (Cat # 2716, Decon Laboratories Inc., King of Prussia, PA, USA) were used in this study, aligning with the BAC in chronic alcohol users and/or those diagnosed with AUD. The AT2 cells were treated with 22, 65, and 130 mM for 6 h to determine concentration-dependent EtOH toxicity. For the time-dependent studies, the cells were treated with 65 mM EtOH for 6, 24, and 72 h. Our preliminary study revealed an approximate 30% reduction in EtOH concentration within the culture medium over a 24 h incubation period. Therefore, for the 72 h treatment, the EtOH concentration was replenished every 24 h to maintain consistent exposure levels throughout the experiment. However, due to practical feasibility considerations and the need to investigate progressive cellular changes in response to EtOH exposure, only a subset of experiments was conducted for the time-dependent studies.

### 2.3. Characterization of Immortalized Human AT2 Cells and Cytotoxicity of EtOH

Morphology and phenotype of the AT2 cells were assessed by staining the cells with LysoTracker Green DND-26 (Cat # L7526, ThermoFisher Scientific, Houston, TX, USA) according to the manufacturer’s instructions. The LysoTracker Green DND-26 is a cell-permeable, non-fixable, green, fluorescent dye that stains acidic compartments and selectively accumulates in the lamellar bodies of AT2 cells [43]. The Green DND-26 was visualized using an Olympus IX71 microscope equipped with a FITC filter at an excitation/emission maxima of 504/511 nm.

Additionally, an expression of surfactant protein-C (SP-C), a functional marker for AT2 cells, was evaluated by immunofluorescence staining using an antibody against SP-C [35,40,44]. In brief, the cells were fixed in ice-cold methanol for 5 min at room temperature and washed thrice with 1X phosphate-buffered saline (PBS, pH 7.4). Non-specific binding was blocked with 1% bovine serum albumin prepared in PBS and Tween-20. After a series of washings, the cells were incubated with primary antibodies to SP-C (1:25 dilution, Cat# PA5-71680, ThermoFisher Scientific, Houston, TX, USA) overnight at 4 °C followed by incubation with Goat anti-Rabbit IgG (H+L), secondary antibodies, DyLight™ 488 (1:500 dilution, Cat# 35552, ThermoFisher Scientific, Houston, TX, USA) for 1 h at room temperature in the dark. The counterstaining was done with 4′,6-diamidino-2-phenylindole (DAPI, Cat # 135-1303, Bio-Rad Laboratories, Hercules, CA, USA). Fluorescence was visualized under Olympus IX73 microscope equipped with a GFP filter, and the fluorescence intensity was quantified using NIH ImageJ 1.50r.

The viability of the AT2 cells treated with EtOH was determined using the Calcein AM cell viability assay kit (Cat # 30026, Biotium, Inc., Fremont, CA, USA) as per the manufacturer’s instructions. The AT2 cells were seeded in a 96-well plate and incubated with various concentrations of EtOH for 6 h. After aspirating the culture medium, 100 µL of 2 µM Calcein AM in PBS was added to the wells and incubated at 37 °C for an additional 30 min. The fluorescence, measured at 485/530 nm excitation/emission wavelengths using Bio-Tek Synergy/HTX multi-mode reader (Winooski, VT, USA), was expressed as a percent of the controls.

Lactate dehydrogenase (LDH), a cytosolic protein widely used as a marker of cytotoxicity [45,46] was assayed in culture media of the cells treated with EtOH using the LDH-Glo cytotoxicity assay kit (Cat# J2380, Promega Corporation, Madison, WI, USA). The luminescence was measured using a Bio-Tek Synergy/HTX multi-mode reader (Winooski, VT, USA), and the amount of LDH released into the medium is expressed as a percentage of the cytotoxicity.

### 2.4. Cytokines and Chemokines

Secreted cytokines and chemokines in culture media from EtOH-treated AT2 cells were quantified using a Human Cytokine Antibody Array kit (Cat # ab133996, Abcam, Cambridge, MA, USA). Briefly, an aliquot of the culture media was incubated on the array membrane, and the target cytokines and chemokines were visualized using the chemiluminescence detection system. The signal intensity for each cytokine and chemokine spot was quantified using NIH ImageJ 1.50r. The data normalized to the controls and expressed as arbitrary units (a.u.).

### 2.5. Oxidative Stress

CellROX Orange oxidative stress reagent (Cat # C10443, ThermoFisher Scientific, Houston, TX, USA), a novel fluorogenic probe used to measure oxidative stress. In the reduced state, the dye is non-fluorescent but exhibits bright orange fluorescence upon oxidation by reactive oxygen species (ROS), with an absorption/emission maximum of ∼545/565 nm, respectively. In brief, after the incubation, the culture media of the controls and the EtOH- treated cells were replaced with CellROX Orange reagent at a final concentration of 5 µM, and the cells were incubated at 37 °C for an additional 30 min. The culture medium was removed, and the cells were washed with 1X PBS thrice, followed by nuclear counterstaining using NucBlu Live ReadyProbe Reagent (Cat # R37605, ThermoFisher Scientific, Houston, TX, USA). The fluorescent images were acquired using an Olympus IX73 microscope equipped with a TRITC filter, and the relative fluorescence was quantified by ImageJ.

### 2.6. Mitochondrial Stress and Real-Time ATP Production Rate

The mitochondrial stress and real-time ATP production rate in AT2 cells treated with EtOH were determined by Seahorse XFp extracellular flux analyzer (Agilent, Santa Clara, CA, USA) [35,47,48]. In brief, ~40,000 cells/well were seeded into Seahorse XFp cell culture miniplates, followed by treatment with 22 and 65 mM EtOH. These EtOH concentrations are reported in alcoholic subjects [41]. A concentration of 130 mM EtOH was excluded from bioenergetic analyses due to its high cytotoxicity, which could compromise and interfere with accurate metabolic measurements.

For the assay, the cell culture medium was changed to Dulbecco’s modified Eagle’s medium (DMEM, pH 7.4) supplemented with 10 mM Glucose, 2 mM L-glutamine, and 2 mM pyruvate, followed by incubation in a non-CO_2_ incubator at 37 °C for 60 min, necessary for degassing the plate and allowing CO_2_ to diffuse [35,47,48].

The mitochondrial respiratory function “stress” test protocol was used to measure indices of mitochondrial function in cells treated with EtOH, as per the manufacturer’s instructions. Oligomycin, trifluoromethoxy carbonylcyanide phenylhydrazone (FCCP); antimycin A, and rotenone were injected sequentially through ports of the Seahorse Flux cartridges to achieve final concentrations of 2 µM, 1 µM, and 0.5 µM, respectively, to measure the basal oxygen consumption rate (OCR), oxygen consumption linked to ATP production, non-ATP-linked oxygen consumption (proton leak), maximal and spare respiration capacity, and finally non-mitochondrial oxygen consumption [35,47,48].

The cellular ATP production rate was determined using a real-time ATP rate assay (Cat # 103591-100, Agilent, Santa Clara, CA, USA), which measures total cellular ATP production rate from the two main metabolic pathways, oxidative phosphorylation (OXPHOS), and glycolysis. A metabolic modulator (oligomycin and a mixture of rotenone and antimycin A) was serially injected, allowing for calculation of in situ mitochondrial and glycolytic ATP production rates in the live cells treated with/without EtOH. Oligomycin inhibits mitochondrial ATP synthesis, resulting in a decreased oxygen consumption rate (OCR), allowing the mitochondrial ATP production rate to be quantified. Extracellular acidification rate (ECAR) data combined with the buffer factor of the assay medium (2.5 mmol/L/unit of pH) to allow the calculation of total proton efflux rate (PER). A complete inhibition of mitochondrial respiration, achieved with rotenone plus antimycin A, accounts for the mitochondria-associated acidification and the glycolytic ATP production rate, as observed in combination with PER data. The test was performed by injecting oligomycin and antimycin A/rotenone mix through ports of the Seahorse Flux cartridges to provide final concentrations of 1.5 µM and 0.5 µM, respectively, to measure the total, mitochondrial, and glycolytic ATP rates [35,47,48].

### 2.7. Analysis of Fatty Acid Ethyl Esters (FAEEs) and Dipalmitoylphosphatidylcholine (DPPC)

FAEEs were analyzed in AT2 cells treated with EtOH as described previously [49]. In brief, a mix of 9 deuterium labelled FAEEs; ethyl laurate-d5 (12:0, Cat # TRC-E917387), ethyl myristate-d5 (14:0, Cat # TREC-E925182), ethyl palmitate-d5 (16:0, Cat # TREC-E925482), ethyl pamitoleate-d5 (16:1, Cat # TREC-E925857), ethyl stearate-d5 (18:0, Cat # TREC-E925952), ethyl oleate-d5 (18:1, Cat # TREC-E925327), ethyl linoleate-d11 (18:2, Cat # 37973), ethyl linolenate-d5 (18:3, cat # 28185) and ethyl arachidonate-d11 (20:4, Cat # 37970) obtained from LGC Standards, Manchester, NH, USA, and Cayman Chemical as an internal standard were added into the culture flask at the end of incubation. After mixing, the cells along with incubation media were transferred to glass tubes, extracted with chloroform: methanol (2:1, *v*/*v*), dried under nitrogen, and reconstituted with 70 µL dichloromethane: methanol (1:1 *v*/*v*) [49]. The reconstituted samples were analyzed by reverse phase liquid chromatography on a Kinetex C18 HPLC column (2.6 µm, 150 × 2.1 mm, Phenomenex) with the following set conditions-100% mobile phase A: water + 0.1% formic acid to 100% mobile phase B: acetonitrile + 0.1% formic acid at a flow rate of 0.450 mL/min for 10 min. The eluted analytes were detected by MS/MS using positive electrospray ionization (ESI). Source and gas settings were as follows: Curtain Gas  =  30 psi, CAD Gas  =  Medium, Ion Spray Voltage  =  5.5  kV, and Temperature  =  350  °C. Liquid chromatography–mass spectrometry (LC-MS/MS) was performed using an Acquity Premier UHPLC System (Waters) coupled to a Turbo V electrospray ionization source and a Qtrap 6500 mass spectrometer (SCIEX).

Chromatographic peaks were integrated using MultiQuant software 3.0 (SCIEX), and retention times were manually inspected, corrected, and normalized by the peak areas of internal standards. External calibration curves prepared from FAEE standards [12:0, 14:0, 16:0, 16:1, 18:0, 18:1, 18:2, 18:3, and 20:4 FAEEs (Cat # 10008188, Cayman Chemical, Ann Arbor, MI, USA)] were used to quantify individual FAEE and expressed as ng/10^6^ cells.

DPPC, a major phospholipid component of pulmonary surfactants, is produced and secreted by the AT2 cells in the lungs. Therefore, the levels of DPPC were measured in the AT2 cells treated with EtOH by LC-MS/MS. In brief, the treated AT2 cells along with culture medium were transferred into glass tubes, mixed with deuterium labelled DPPC as an internal standard (UltimateSPLASH ONE, Cat # 330820, Avanti Research, Alabaster, AL, USA), followed by extraction of lipids with chloroform: methanol (2:1, *v*/*v*) [49]. The extract was dried under a gentle stream of nitrogen, and the dried residue redissolved in acetonitrile and analyzed by LC-MS/MS using an Acquity Premier UHPLC System (Waters) coupled to a Turbo V electrospray ionization source and a Qtrap 6500 mass spectrometer (SCIEX).

DPPC was analyzed using hydrophilic interaction liquid chromatography (HILIC) and an amino-capped Luna NH_2_ HPLC column (3 µm, 150 × 4.6 mm, Phenomenex). Mobile phase A: acetonitrile/water/hexane (92:6:2, vol/vol/vol) + 10 mM ammonium acetate, pH 9.3, and mobile phase B: acetonitrile/water (50:50, vol/vol) + 10 mM ammonium acetate, pH 9.3 were used. The gradient was as follows: 0–2 min, 100% A; 2–6.5 min, 100% to 60% A; 6.5–7 min, 60% to 55% A; 7–9 min, 55% A; 9–9.5 min, 55% to 30% A; 9.5–12 min, 30% A; 12–12.1 min, 30% to 15% A; 12.1–14.5 min, 15% A; 14.5–14.6 min, 15% to 0% A; 14.6–17 min, 0% A; 17–18.1 min, 0% to 100% A; 18.1–22 min, 100% A. The flow rate was 1 mL/min, the column temperature was 35 °C, and the injection volume was 5 µL. Lipids were analyzed using scheduled multiple reaction monitoring (sMRM) and the ESI source parameters set as follows: CUR = 30 psi, CAD gas = medium; temperature = 450 °C, GS1 = 50 psi; GS2 = 55 psi, and Ion Spray Voltage = 5.5 kV in positive ionization mode and −4.5 kV in negative ionization mode.

Electrospray ionization (ESI) with positive/negative polarity switching and sMRM was used for MS/MS detection of the target ions following the source and gas settings as follows: Curtain Gas  =  35, CAD Gas  =  medium, temperature  =  550  °C. Ion Spray Voltage = 5.5 kV in positive ionization mode and −4.5 kV in negative ionization mode.

The peak area for DPPC was normalized to the corresponding peak areas of the internal standard. Analyst software (V. 1.7, SCIEX) operated LC-MS instrument and acquired the data. Skyline software 22.2 (RRID: SCR_014080) was used for peak picking and exporting sample data. Multivariate statistical analysis was done by Metaboanalyst 5.0, which supports processing of raw MS spectra, comprehensive data normalization, and statistical, functional, and meta-analyses.

### 2.8. Western Blot Analysis

As described earlier, the AT2 cells treated with EtOH were harvested followed by lysis using 1X RIPA buffer (Cat# 9806, Cell Signaling Technology) containing phosphatase and protease inhibitors. Expression of alcohol-metabolizing enzymes and key up- and downstream proteins involved in AMPKα and ER stress signaling was assayed by Western blot analysis using respective antibodies [35,47]. Briefly, protein concentration was measured in the cell lysates using Bio-Rad protein assay (Cat # 5000006, Bio-Rad Laboratories, Hercules, CA, USA). An equal amount of the lysate was mixed with sample buffer and boiled for 10 min, and ~40 μg of protein was subjected to SDS PAGE, protein bands transferred onto PVDF membrane, which was incubated with secondary antibodies, and the protein bands developed with an enhanced chemiluminescence (ECL) detection reagent (ThermoFisher Scientific, Houston, TX, USA) as described previously [35,47]. The protein band intensities were determined and compared using ImageJ.

### 2.9. Quantitative Real-Time Polymerase Chain Reaction (qPCR) Analysis

Total RNA was extracted from EtOH-treated AT2 cells using a Quick-RNA Miniprep kit (Cat # R1054, Zymo Research, Tustin, CA, USA) according to the manufacturer’s instructions. The purity of extracted RNA was verified by absorbance ratios at 260/280 nm and 260/230 nm using a BioTek Epoch 2 Take 3 microvolume plate reader. Total RNA (500 ng) was reverse-transcribed to cDNA using the iScript cDNA Synthesis Kit from Bio-Rad Laboratories (Bio-Rad# 1708891). The primers for qPCR analysis were designed using the Primer Quest tool obtained from Integrated DNA Technologies, Inc., Coralville, IA, USA. The qPCR analysis was performed using Applied Biosystems Quant Studio 3 Real-Time PCR System and Powerup SYBR Green Master mix protocol (Cat# A25741, Waltham, MA, USA) with the primer set described in Table 1. Amplification efficiencies were normalized using the housekeeping gene *Rplpo*. The relative fold change in gene expression was calculated by the Livak method [50].

### 2.10. Statistical Analysis

One-way ANOVA was used to analyze the data sets, followed by the Tukey Multiple Comparison test for statistical significance. The values are expressed as Mean ± SEM (standard error of mean) using GraphPad Prism (v7.0, San Diego, CA, USA). The statistical significance was set at a *p*-value ≤ 0.05.

## 3. Results

### Phenotypic Characterization of Immortalized Human AT2 Cells

**Figure 1:**
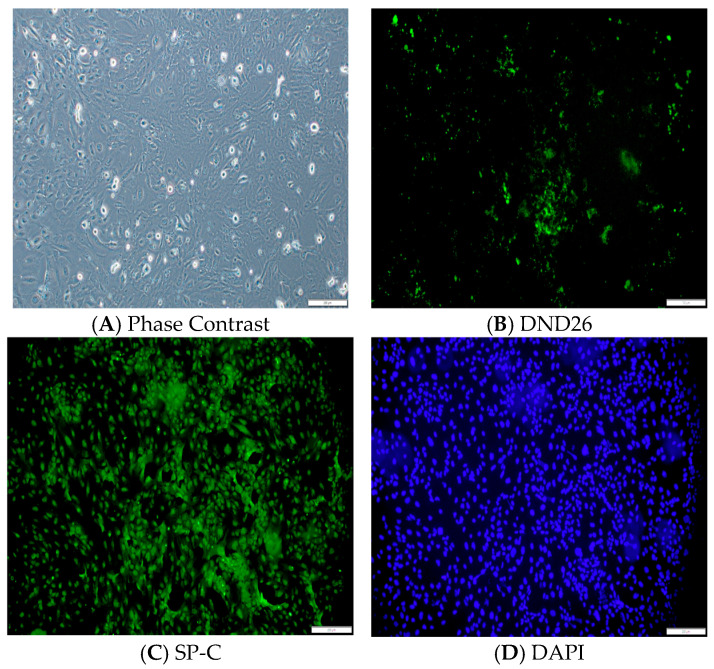
Morphology and Characterization of AT2 cells. Phase-contrast image showing AT2 cell morphology (**A**), lysotracker Green DND-26 fluorescence indicates lamellar bodies characteristic of AT2 cells (**B**), immunofluorescence staining of surfactant protein C (SP-C) confirms AT2 phenotype (**C**), and nuclear staining DAPI (**D**). Magnification 10X, scale 200 µm.

As shown in Figure 1, immortalized human AT2 cells maintained phenotypic and physiological characteristics of primary human AT2 cells throughout the study. A green fluorescence of LysoTracker Green DND-26, along with significant expression of SP-C, a functional marker of AT2 cells, confirmed their phenotypic characteristics during the culture period.

## 4. Concentration-Dependent Studies

### 4.1. Viability and Cytotoxicity

**Figure 2:**
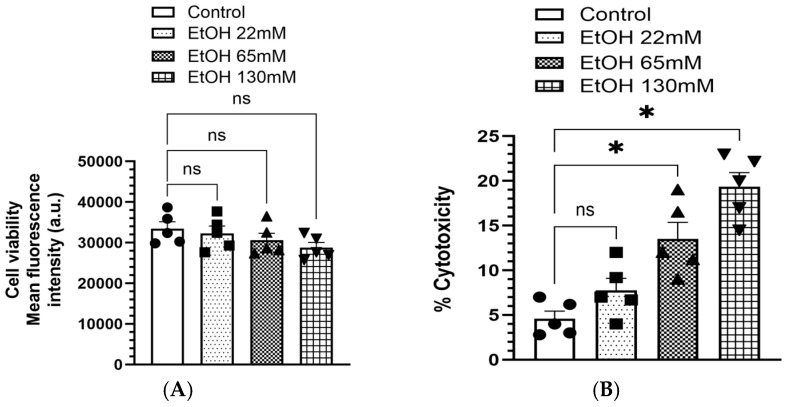
Cell viability (**A**) and Cytotoxicity (**B**) in AT2 cells treated with 22, 65 and 130 mM of EtOH for 6 h. Values are expressed as Mean ± SEM (*n* = 5 replicates). * *p*-value ≤ 0.05 compared to the controls. ns—non-significant.

Treatment of immortalized human AT2 cells with 22–130 mM EtOH for 6 h did not significantly alter overall cell viability (Figure 2A). However, EtOH exposure produced a concentration-dependent increase in lactate dehydrogenase (LDH) release, indicating membrane damage and cytotoxicity. Specifically, LDH activity in the culture medium increased approximately 3- and 4-fold in cells treated with 65 and 130 mM EtOH, respectively, compared with control (Figure 2B). In contrast, cells treated with 22 mM EtOH showed no significant change in LDH release relative to the controls.

### 4.2. EtOH-Induced Secretion of Cytokines and Chemokines

**Figure 3:**
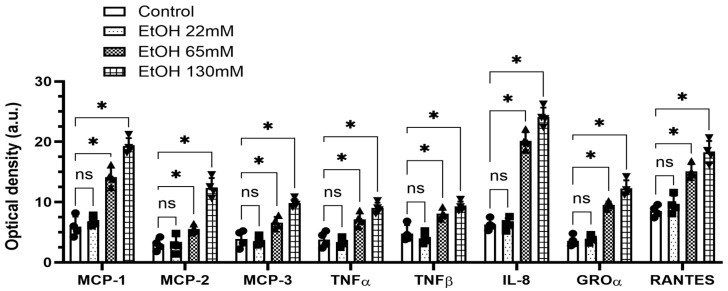
Cytokines/chemokines secreted into the culture medium from AT2 cells treated with 22, 65, and 130 mM EtOH for 6 h. The optical density of 8 detected cytokines and chemokines (out of 23 analyzed) was determined. Values are expressed as Mean ± SEM (*n* = 4 replicates). * *p*-value ≤ 0.05 compared to the controls. ns—non-significant. MCP—Monocyte Chemoattractant Protein; TNF—tumor necrosis factor; IL—Interleukin; GROα—Growth Regulated Oncogene α; RANTES-Regulated upon Activation, Normal T-cell Expressed and Secreted.

Among the 23 key human cytokines and chemokines assayed in the culture medium of AT2 cells treated with EtOH (65 and 130 mM), 8 prominent inflammatory cytokines and chemokines detected were significantly increased (Figure 3). Levels of secreted cytokines and chemokines were estimated to be ~2 and 4-fold higher from the cells treated with 65 and 130 mM EtOH, respectively, than those from the control cells. However, similar cytokines and chemokines were not significantly increased in the culture medium of cells treated with 22 mM EtOH.

### 4.3. EtOH-Induced Oxidative Stress

**Figure 4:**
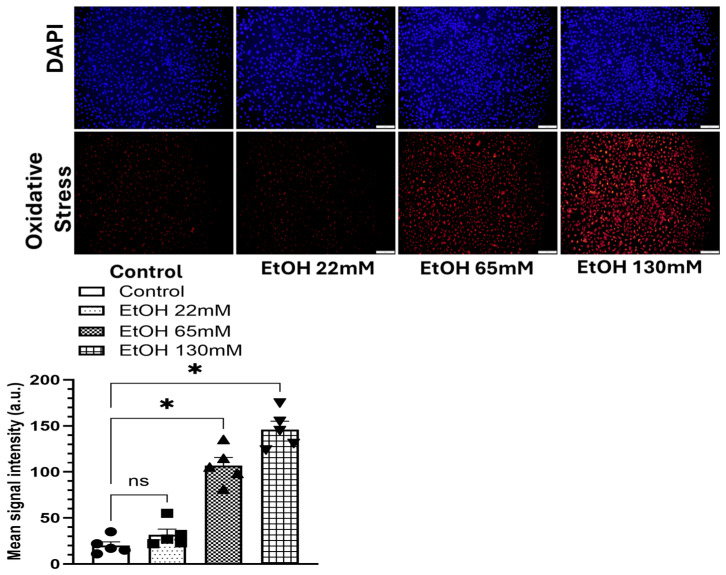
Oxidative stress in AT2 cells treated with 22, 65, and 130 mM EtOH for 6 h. DAPI -upper panel, CellROX orange dye fluorescence-lower panel. Quantification of fluorescence intensity using ImageJ is shown as a bar graph. Values are expressed as Mean ± SEM (*n* = 5 replicates). * *p*-value ≤ 0.05 compared to the respective controls. ns—non-significant. Magnification 10X, scale 200 µm.

As shown in Figure 4, EtOH exposure induced a concentration-dependent oxidative stress in AT2 cells, as evidenced by enhanced fluorescence intensity of the CellROX™ reagent. Fluorescence intensity increased ~5 and 7-fold in AT2 cells treated with 65 and 130 mM EtOH, respectively, compared to the controls, indicating significant generation of reactive oxygen species (ROS). Interestingly, cells treated with 22 mM EtOH showed no measurable increase in oxidative stress relative to the controls.

### 4.4. EtOH-Induced Mitochondrial Stress and Reduced Real-Time ATP Production Rate

**Figure 5:**
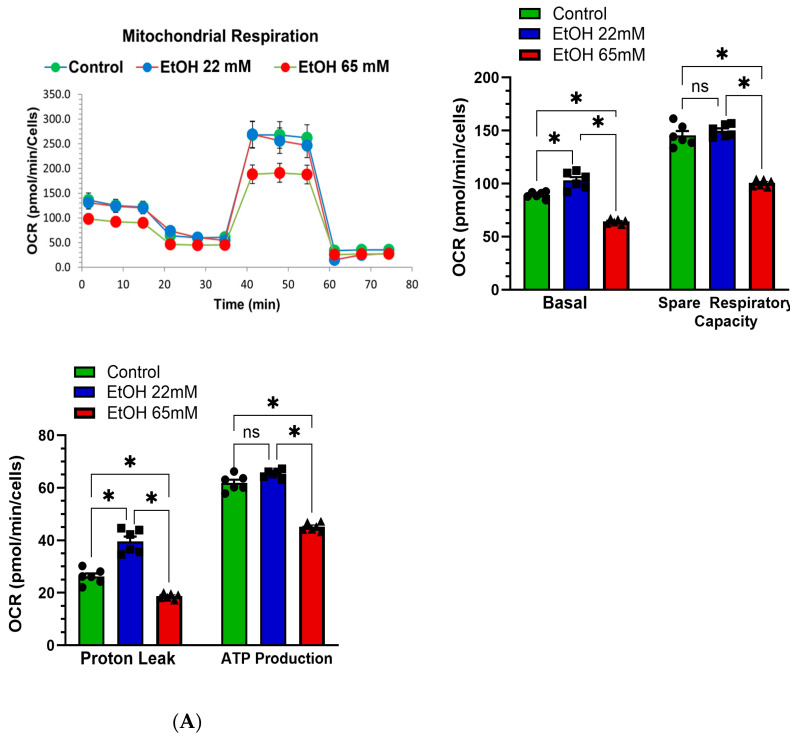

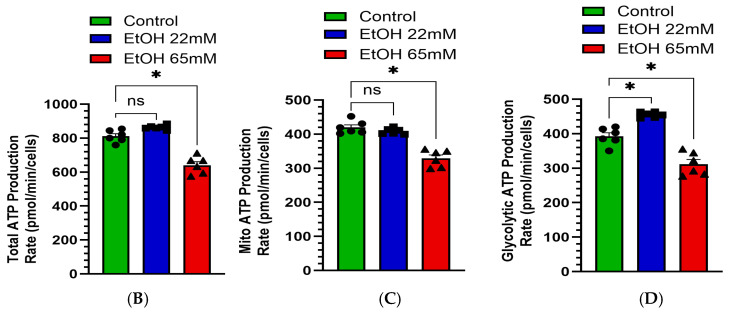
Mitochondrial respiration and bioenergetics (**A**) and real-time ATP production rate (**B**–**D**) in AT2 cells treated with 22 and 65 mM EtOH for 6 h using the Seahorse extracellular flux analyzer. The Mito stress test reveals decreased real-time mitochondrial basal oxygen consumption rate (OCR), spare respiratory capacity (SRC), and mitochondrial ATP production. Real-time ATP production rate data shows reduced total (**B**), mitochondrial (**C**), and glycolytic (**D**) ATP production rates. Values are expressed as Mean ± SEM (*n* = 3 independent experiments). * *p*-value ≤ 0.05 compared to the respective controls. ns—non-significant.

As shown in Figure 5A, AT 2 cells treated with 65 mM EtOH showed a significant reduction in basal oxygen consumption rate (OCR) from ~88 to 64 pmol/min, and spare respiratory capacity (SRC, an index of mitochondrial function) from ~146 to 100 pmol/min. Similarly, the mitochondrial ATP production rate was significantly reduced by approximately 1.3-fold in AT2 cells treated with 65 mM EtOH (~45 pmol/min) compared to the control cells (~62 pmol/min). In contrast, treatment with 22 mM EtOH did not significantly impair mitochondrial function in the AT2 cells. Rather, there was a significant increase for basal OCR (~105 pmol/min) and proton leak (~39 pmol/min), and a moderate increase for SRC (~149 pmol/min), and mitochondrial ATP production rate (~66 pmol/min) relative to controls.

As shown in Figure 5B, the AT2 cells treated with 65 mM EtOH, the total ATP production rate was significantly reduced from ~810 to 640 pmol/min. These cells also showed a significant reduction in both mitochondrial ATP production rate from ~420 to 330 pmol/min (Figure 5C), as well as glycolytic ATP production rate from ~390 to 311 pmol/min (Figure 5D). Of importance, the glycolytic ATP production rate was significantly increased in AT2 cells treated with 22 mM EtOH (~455 pmol/min) as compared to the controls (~390 pmol/min) (Figure 5C). However, no significant changes were observed in total, and mitochondrial ATP production rates for the cells treated with 22 mM EtOH (Figure 5B–D).

### 4.5. Expression of EtOH Metabolizing Proteins and Genes

**Figure 6:**
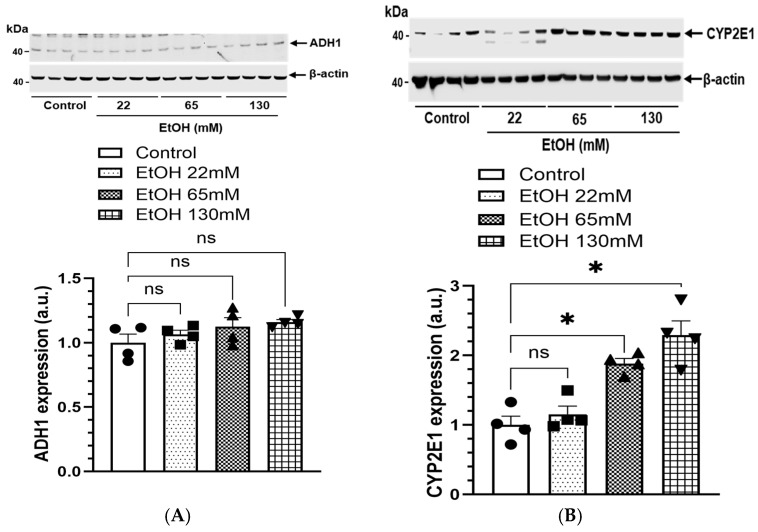

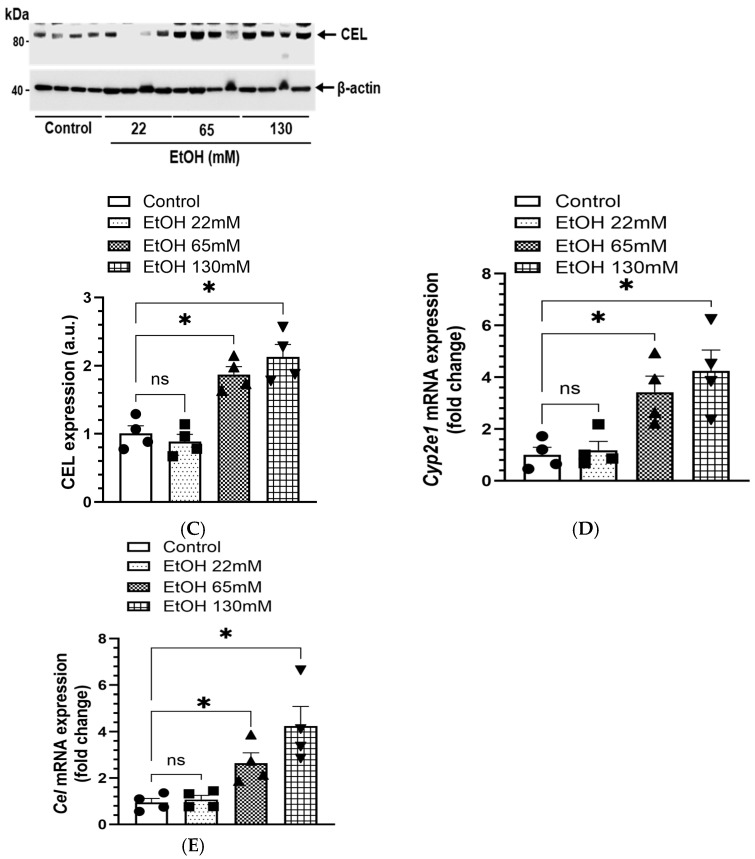
Representative immunoblots and corresponding bar graphs showing protein expression of ADH1 (**A**), CYP2E1 (**B**) and CEL (**C**) in AT2 cells treated with 22, 65, and 130 mM EtOH for 6 h. Intensities normalized to β-actin (loading control). mRNA expression of *Cyp2e1* (**D**) and *Cel* (**E**) in AT2 cells treated with 22, 65, and 130 mM EtOH for 6 h. The gene expression was normalized to Rplpo (housekeeping gene). ADH—Alcohol dehydrogenase; CYP2E1—Cytochrome P450 2E1; CEL—Carboxyl ester lipase. Values are expressed as Mean ± SEM (*n* = 4 replicates). * *p*-value ≤ 0.05 compared to the respective controls. ns—non-significant.

The expression of enzymes involved in oxidative EtOH metabolism (catalyzed by ADH1 and CYP2E1), and non-oxidative EtOH metabolism (catalyzed by CEL, involved in the synthesis of FAEEs) in AT2 cells is shown in Figure 6. Expression of ADH1 was not induced at all in the cells treated with either 22, 65, or 130 mM EtOH (Figure 6A), suggesting a limited contribution of ADH1 in the oxidative metabolism of alcohol in the lungs. However, significant concentration-dependent increases for CYP2E1 (Figure 6B) and CEL (Figure 6C) were observed in AT2 cells treated with 65 and 130 mM EtOH. Of note, a ~2-to-4-fold increase for the mRNA expression of *Cyp2e1* (Figure 6D) and *Cel* (Figure 6E) observed in the cells treated with 65 and 130 mM EtOH, respectively. Therefore, role of CYP2E1 and CEL in the oxidative and nonoxidative metabolism of EtOH, respectively, in AT2 cells could contribute to overall alcoholic lung injury.

### 4.6. Increased Formation of FAEEs in AT2 Cells Treated with EtOH

**Figure 7:**
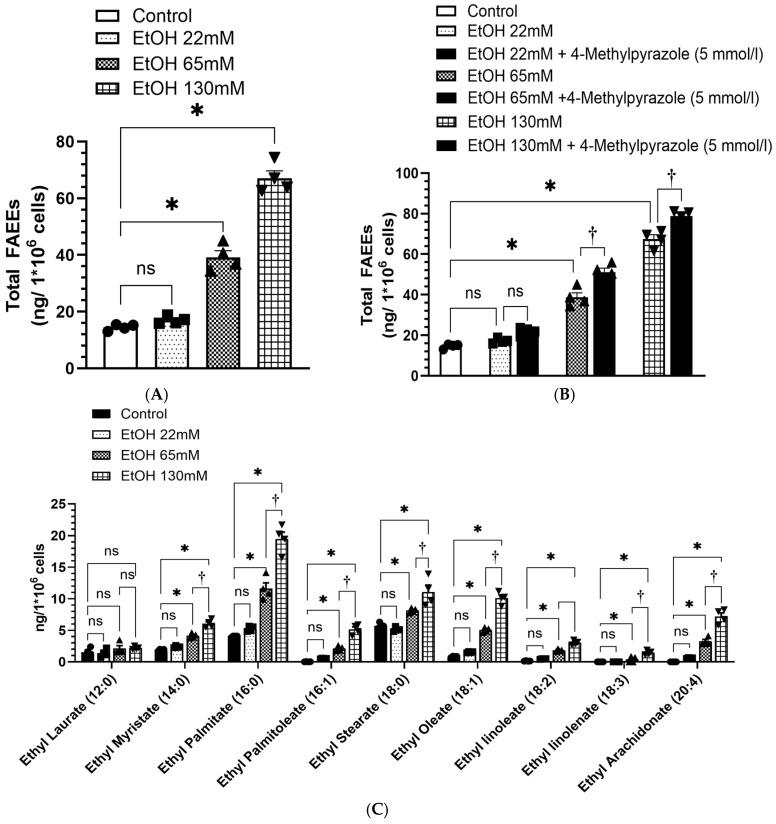
Formation of FAEEs in AT2 cells treated with 22, 65 and 130 mM EtOH for 6 h (**A**), and in the cells simultaneously treated with or without ADH1 inhibitor (4-methylpyrazole) (**B**). Individual FAEE levels in AT2 cells treated with 22, 65 and 130 mM EtOH for 6 h (**C**). Values are expressed as Mean ± SEM (*n* = 4 replicates). * *p*-value ≤ 0.05 compared to the respective controls. ^†^ *p*-value ≤ 0.05 compared to the EtOH 130 mM. ^†^ *p*-value ≤ 0.05 compared to EtOH plus ADH1 inhibitor treated group. ns—non-significant.

As shown in Figure 7A, a concentration-dependent formation of FAEEs in AT2 cells incubated for 6 h with 22, 65, and 130 mM EtOH, averaged to ~17, 39, and 67 ng/1 × 10^6^ cells, respectively. The total FAEE levels were ~3- and ~4-fold greater in the cells treated with 65 and 130 mM EtOH, respectively, as compared to the controls.

Furthermore, inhibition of ADH1 by pretreating the AT2 cells with 4-methylpyrazole (4-MP, 5 mmol/l) for an h, significantly increased formation of FAEEs in the cells treated with 65 mM EtOH (from 38 to 51 ng/1 × 10^6^ cells) and 130 mM EtOH (from 67 to 78 ng/1 × 10^6^ cells), respectively (Figure 7B). Therefore, in addition to low levels of ADH1, its further inhibition facilitates the formation of FAEEs in EtOH-treated AT2 cells.

Quantitation of FAEEs showed ~2-to-12-fold greater levels of ethyl Myristate (14:0), ethyl palmitate (16:0), ethyl palmitoleate (16:1), ethyl stearate (18:0), ethyl oleate (18:1), ethyl linoleate (18:2), ethyl linolenate (18:3) and ethyl arachidonate (20:4) in the cells treated with 65 and 130 mM EtOH as compared to those in the controls (Figure 7C). Additionally, the levels of ethyl esters of 16:0, 16:1, 18:0, 18:1, and 20:4 fatty acids were ~2-fold higher in the cells treated 130 mM EtOH than those treated with 65 mM EtOH (Figure 7C). Significant changes were not observed for the individual FAEEs in the cells treated with 22 mM EtOH as compared to the controls (Figure 7C).

### 4.7. Reduced Levels of DPPC and Associated SPs in AT2 Cells Treated with EtOH

**Figure 8:**
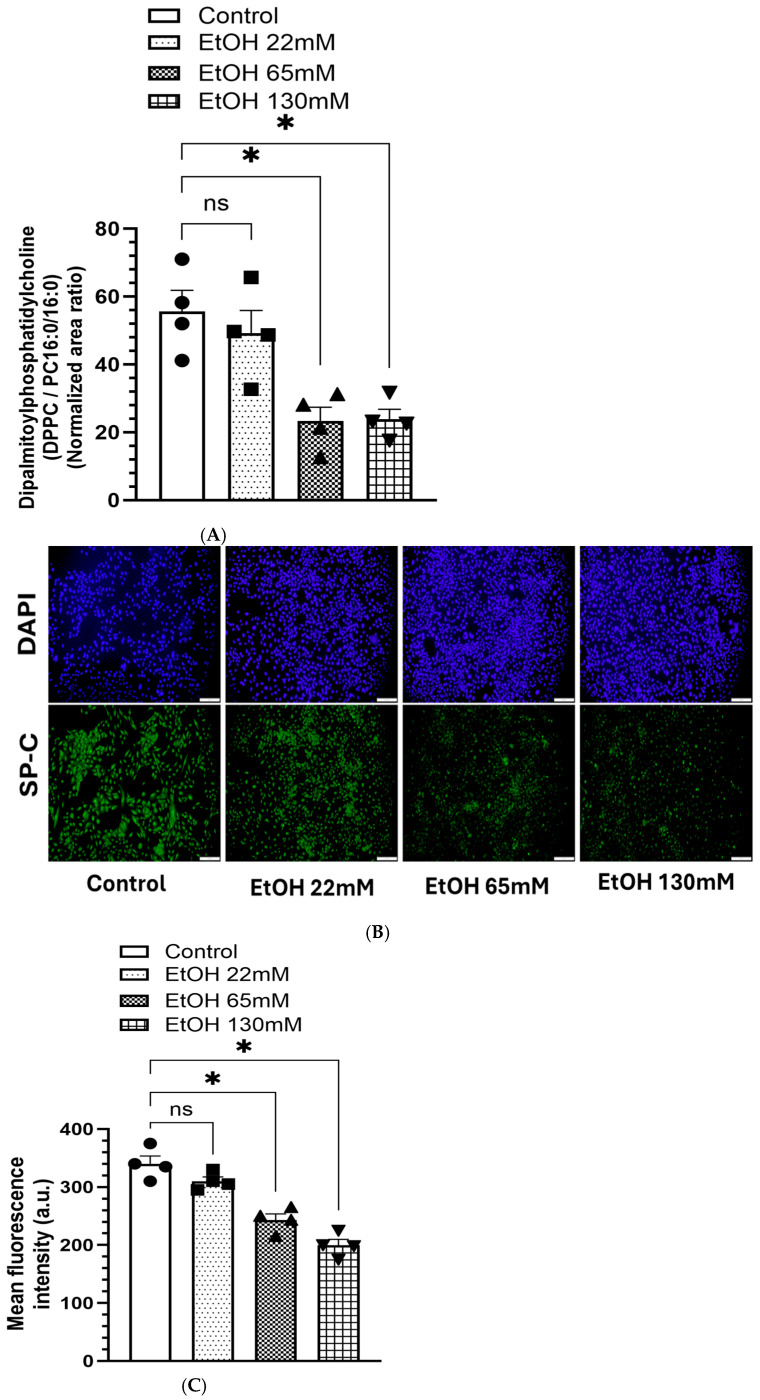
Dipalmitoylphosphatidylcholine (DPPC) levels and expression of surfactant protein C (SP-C) in AT2 cells treated with 22, 65 and 130 mM of EtOH for 6 h. DPPC (**A**). SP-C Immunofluorescence (**B**), Upper Panel-DAPI, Lower Panel–SP-C immunofluorescence. Quantification of fluorescence intensity using ImageJ is shown by the bar graph (**C**). Values expressed as Mean ± SEM (*n* = 4 replicates). * *p*-value ≤ 0.05 compared to the respective controls. ns—non-significant. Magnification 10X, scale 200 µm.

As shown in Figure 8A, a reduced levels of DPPC (a key phospholipid component of the pulmonary surfactants) was observed in AT2 cells treated with 65 and 130 mM EtOH. Similarly, the expression of SP-C was also reduced concentration-dependently in the cells treated with 65 and 130 mM EtOH (Figure 8B,C).

**Figure 9:**
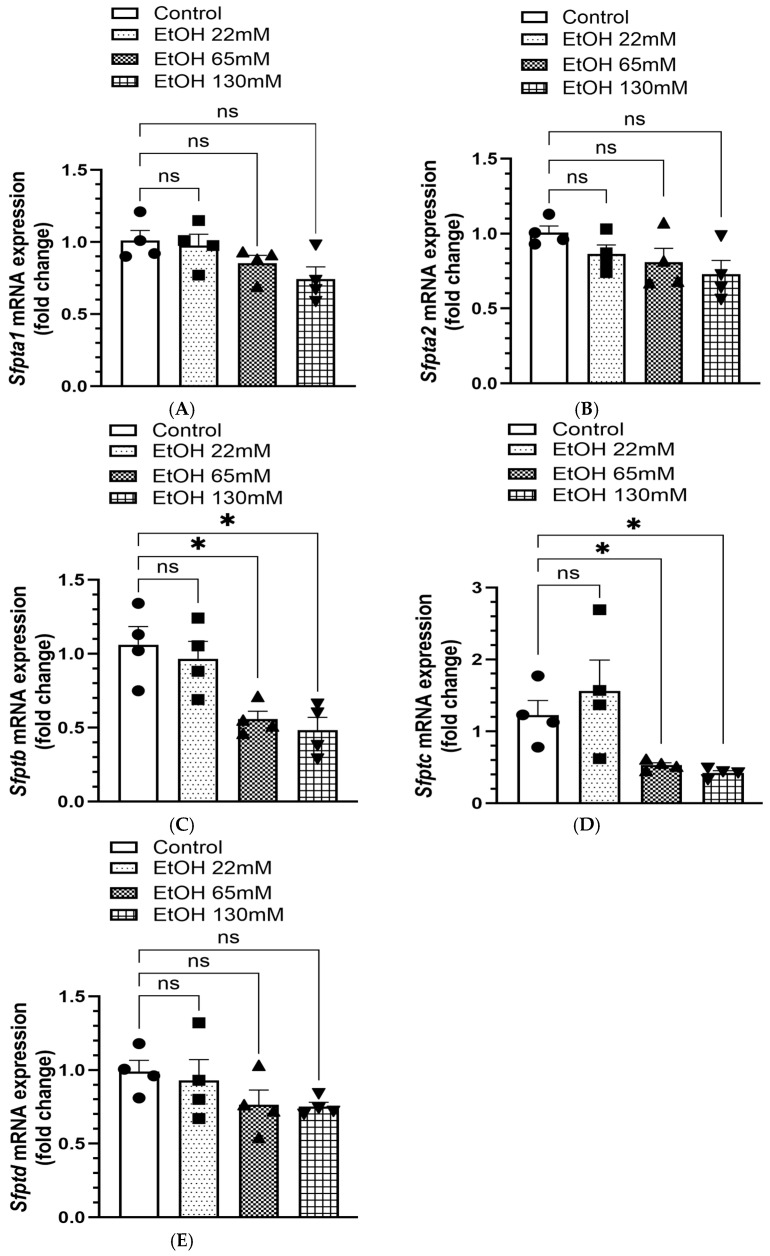
mRNA expression of surfactant proteins (SPs) in AT2 cells treated with 22, 65, and 130 mM EtOH for 6 h. *Sfpta1*, surfactant protein A1 (**A**), *Sfpta2*, surfactant protein A2 (**B**), *Sfptb*, surfactant protein B (**C**), *Sfptc*, surfactant protein C (**D**), and *Sfptd*, surfactant protein D (**E**). The gene expression was normalized to Rplpo (housekeeping gene). Values are expressed as Mean ± SEM (*n* = 4 replicates). * *p*-value ≤ 0.05 compared to the respective controls. ns—non-significant.

The mRNA expression of SP-A1 (*sfpta1*) (Figure 9A), SP-A2 (*sfpta2*) (Figure 9B), and SP-D (*sfptad*) (Figure 9E) was not significantly different in the cells treated with various concentrations of EtOH. However, ~2-to-3-fold decreased mRNA expression of SP-B (*sfptb*) (Figure 9C), and SP-C (*sfptc*) (Figure 9D) were found in the cells treated with 65 and 130 mM EtOH, respectively, compared to the controls.

Overall, significant concentration-dependent decreases for SP-B and SP-C were observed in AT2 cells treated with 65 and 130 mM EtOH, suggesting their key role in dysregulated surfactant homeostasis in the lungs of the subjects with a history of chronic alcohol use/abuse.

### 4.8. EtOH-Induced Changes in AMPKα Signaling and Lipogenesis

**Figure 10:**
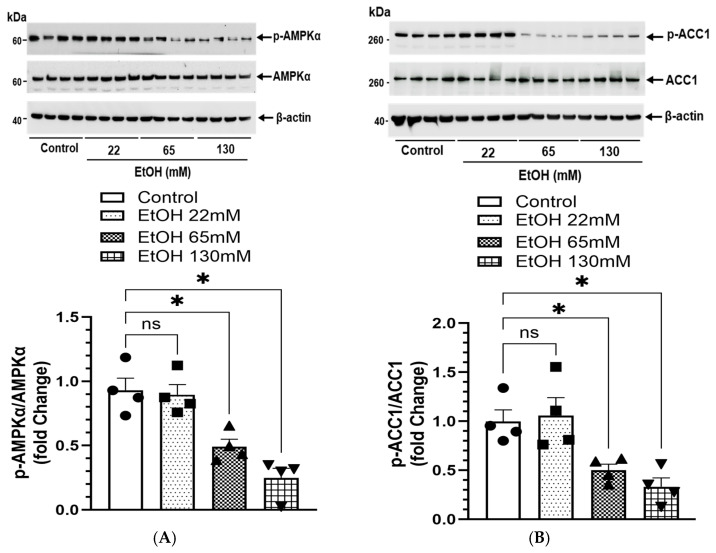

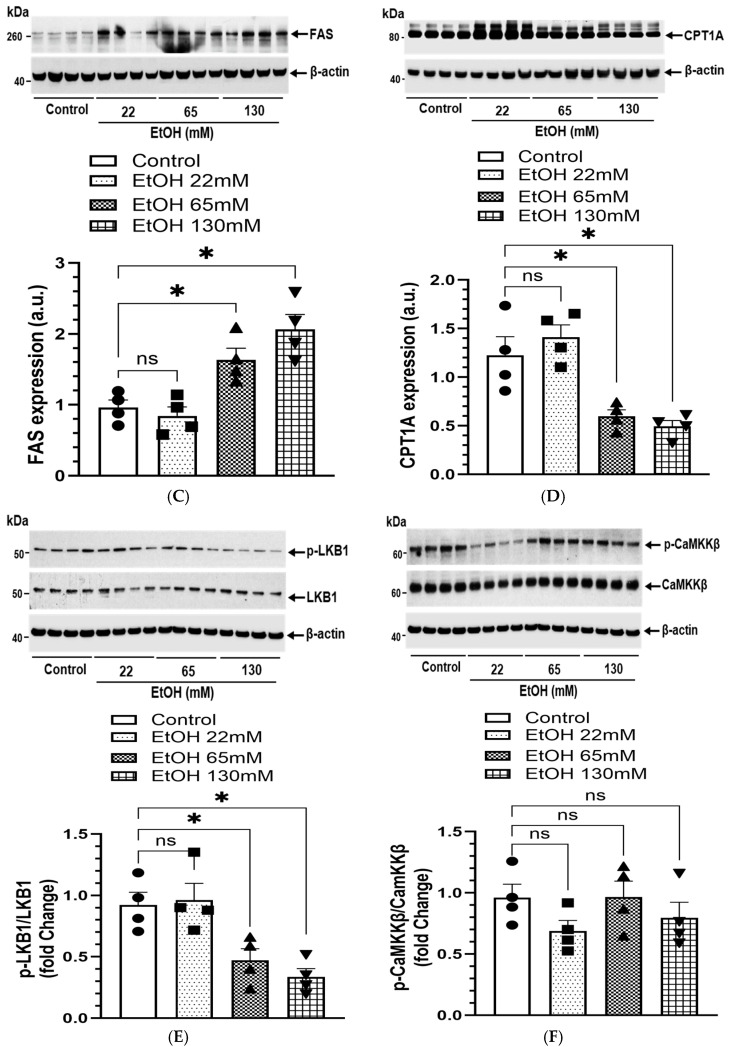
AMPKα signaling in AT2 cells treated with 22, 65, and 130 mM EtOH for 6 h. Representative immunoblots and corresponding bar graphs show protein expression for p-AMPKα/AMPKα (**A**), p-ACC1/ACC1 (**B**), FAS (**C**), CPT1A (**D**), p-LKB1/LKB1 (**E**), and p-CaMKKβ/CaMKKβ (**F**). Intensities normalized to β-actin (loading control). Values are expressed as Mean ± SEM (*n* = 4 replicates). * *p*-value ≤ 0.05 with respect to the controls. ns—non-significant.

As shown in Figure 10A, a concentration-dependent decrease in p-AMPKα levels were found in the AT2 cells treated with 65 and 130 mM EtOH. Concomitantly, an increased expression of key proteins [ACC1 (Figure 10B), FAS (Figure 10C)] involved in lipogenesis was noted in the cells treated with 65 and 130 mM EtOH along with significant concentration-dependent decreases for p-ACC1, a substrate for AMPKα (Figure 10B) and CPT1A, a key protein involved in β-oxidation of fatty acids (Figure 10D). Decreased levels of the AMPKα downstream signaling proteins, p-ACC1 and CPT1A, as mentioned above are strong indicators of increased lipogenesis.

Furthermore, the levels of p-LKB1 (Figure 10E) (upstream kinase regulating activation of AMPKα, oxidative stress, and mitochondrial function) were reduced in a concentration-dependent manner in the cells treated with 65 and 130 mM EtOH. However, no significant changes were observed for p-CaMKKβ (a calcium-binding protein activated by Ca^++^ and calmodulin, sensitive to ER stress) in the cells treated with EtOH (Figure 10F).

In addition, no significant changes in up- and downstream AMPKα signaling were observed in the cells treated with 22 mM EtOH. Overall, these findings suggest that EtOH-induced, inactivation of AMPKα, suppressed its upstream regulator LKB1, decreased CPT1A expression, and increased lipogenesis, as observed in the cells treated with 65 and 130 mM EtOH, could be major contributors to the pathogenesis of ARLD.

### 4.9. EtOH-Induced ER Stress

**Figure 11:**
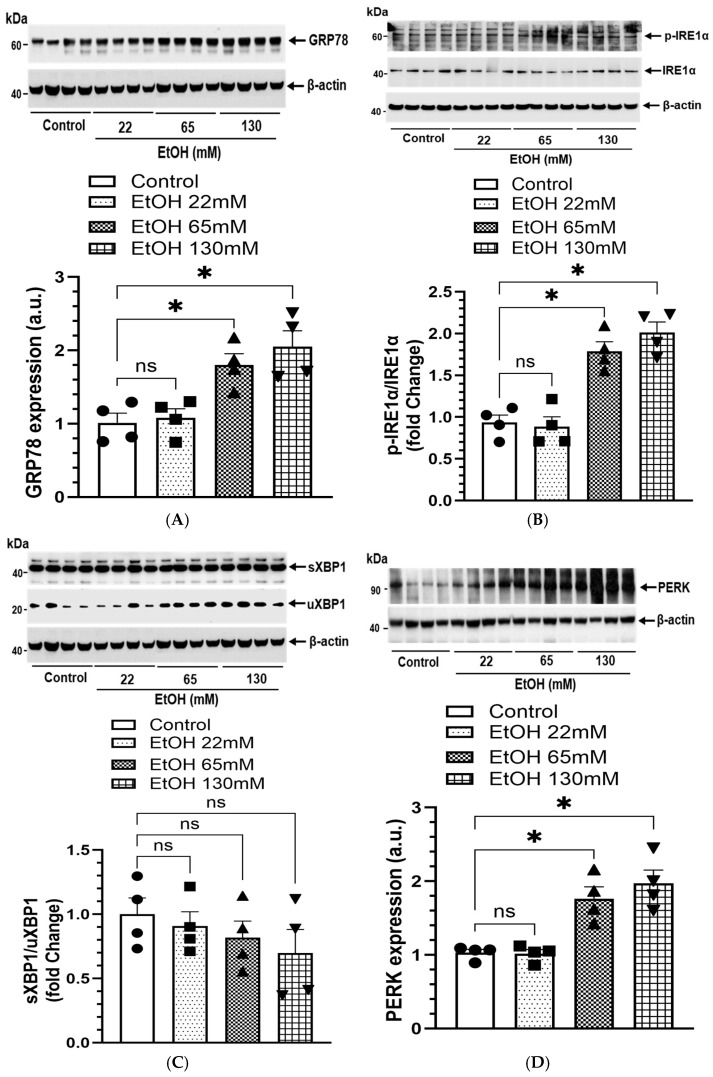

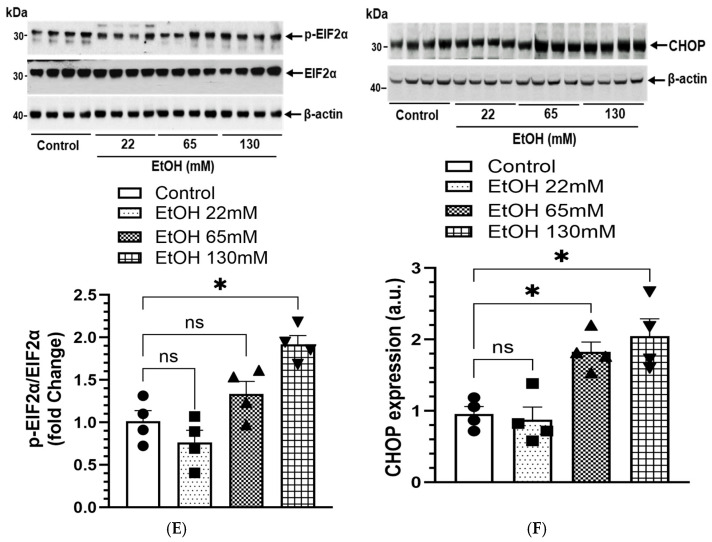
ER stress and UPR signaling in AT2 cells treated with 22, 65, and 130 mM EtOH for 6 h. Representative Immunoblots and corresponding bar graphs show protein expression for GRP78 (**A**), p-IRE1α/IRE1α (**B**), sXBP1/uXBP1 (**C**), PERK (**D**), p-EIF2α/EIF2α (**E**), and CHOP (**F**). Intensities normalized to β-actin (loading control). Values are expressed as Mean ± SEM (*n* = 4 replicates). * *p*-value ≤ 0.05 with respect to the controls. ns—non-significant.

ER stress, as evaluated by the expression of GRP78 and the unfolded protein responses (UPR; p-IRE1α, pEIF2α, PERK, and CHOP), increased concentration-dependently in the AT2 cells treated with EtOH (Figure 11A–F). However, significant changes for spliced XBP1 were not found in EtOH-treated cells (Figure 11C), suggesting that EtOH exposure dysregulates ER homeostasis.

## 5. Time-Dependent Studies

### 5.1. Increased Formation of FAEEs and Expression of CEL in AT2 Cells Treated with EtOH

**Figure 12:**
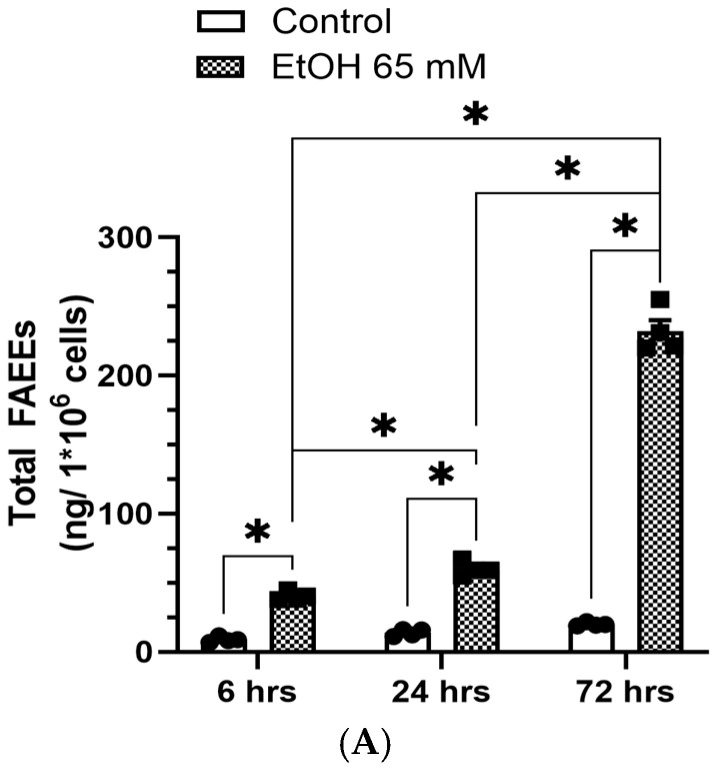

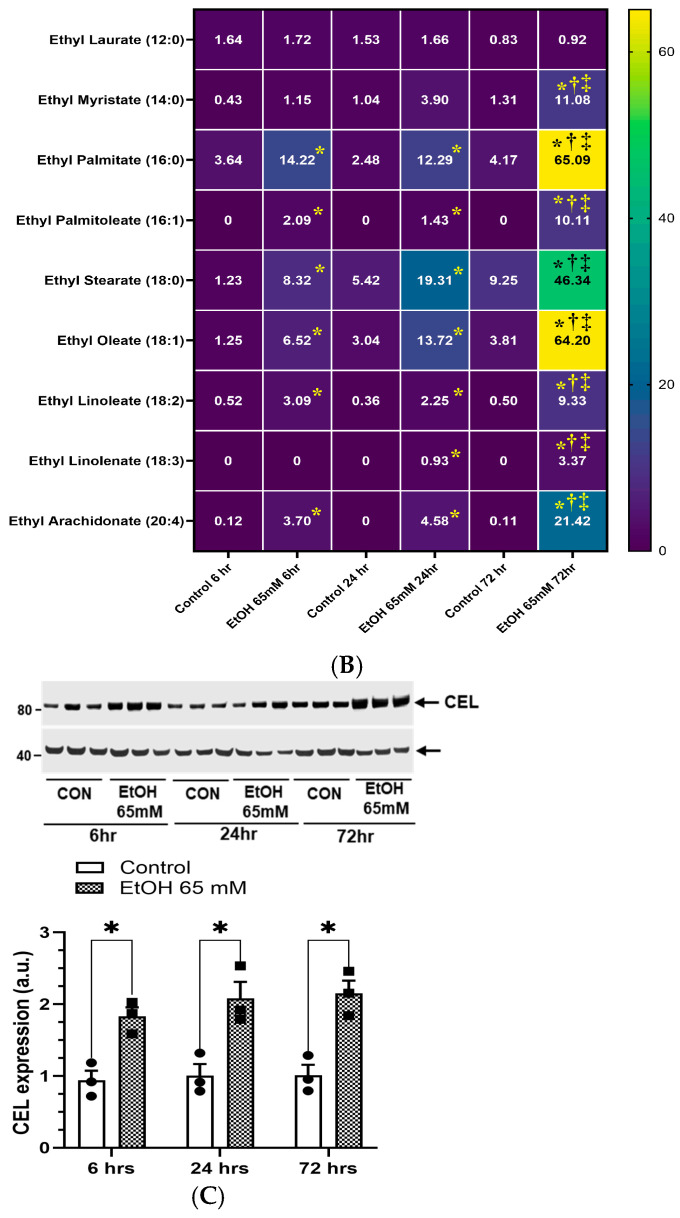
Time-dependent formation of FAEEs and CEL expressions in AT2 cells treated with 65 mM EtOH for 6, 24, and 72 h. Total FAEE levels (**A**). Heatmap showing the mean values of individual FAEE levels determined in AT2 cells treated with 65 mM EtOH for 6, 24, and 72 h (**B**). Immunoblots and corresponding bar graph show protein expression for CEL (**C**). Intensities normalized to β-actin (loading control). Values are expressed as Mean ± SEM (*n* = 4 replicates for FAEEs and *n* = 3 replicates for CEL expression). * *p*-value ≤ 0.05 compared to the controls, † *p*-value ≤ 0.05 compared to 65 mM EtOH 24 h, ‡ *p*-value ≤ 0.05 compared to 65 mM EtOH 6 h.

An increased time-dependent formation of FAEEs was observed in AT2 cells treated with 65 mM EtOH; ~40, 60, and 232 ng/1 × 10^6^ cells at 6, 24, and 72 h, respectively. The formation of FAEEs was ~1.5 to 6-fold greater in the cells treated with 65 mM EtOH for 24 and 72 h, respectively, as compared to those at 6 h. (Figure 12A). Similarly, about ~4-fold increased levels of FAEEs were found in the cells treated with 65 mM EtOH for 72 h, compared to those treated for 24 h.

Furthermore, an increased time-dependent formation of 14:0, 16:0, 16:1, 18:0, 18:1, 18:2, 18:3 and 20:4 FAEEs were found in the cells treated with 65 mM EtOH compared to controls (Figure 12B). About ~3-7-fold increased levels of FAEEs were observed in the cells treated with 65 mM EtOH for 72 h than those for 6 and 24 h, respectively (Figure 12B).

Concomitantly, the expression of CEL was also significantly increased in the AT2 cells treated with 65 mM EtOH for 6, 24 and 72 h (Figure 12C). Although the CEL expression was relatively higher in the cells treated with 65 mM EtOH at 24 and 72 h than at 6 h, it was not statistically significant among the time points studied. Thus, long-term EtOH exposure similarly induced CEL, but formation of FAEEs in EtOH-treated cells increased by several folds with increasing the duration of exposure in this study suggesting the potential of chronic EtOH exposure in the pathogenesis of ARLD.

### 5.2. Reduced DPPC Levels and Expression of SP-C in AT2 Cells Treated with EtOH

**Figure 13:**
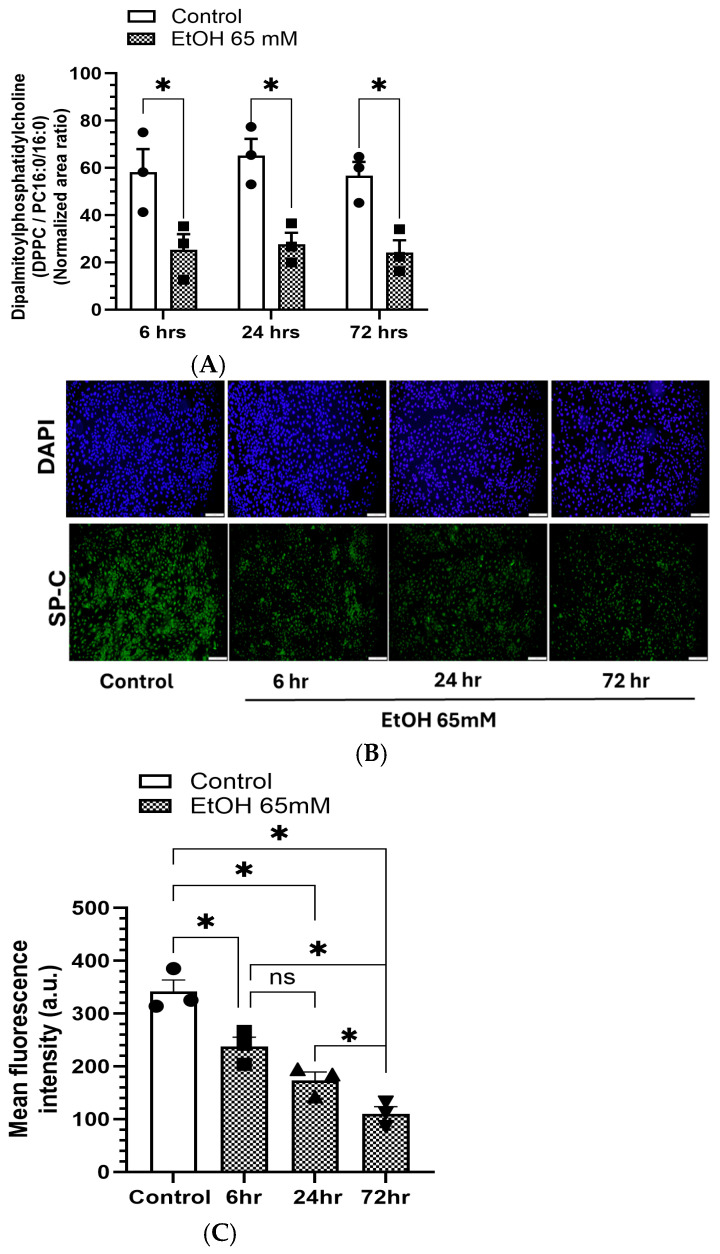
Time-dependent changes in DPPC levels (**A**), and SP-C expression (**B**) in AT2 cells treated with 65 mM EtOH for 6, 24, and 72 h. Upper Panel -DAPI, Lower Panel–SP-C immunofluorescence. Quantification of fluorescence intensity using ImageJ is shown in a bar graph (**C**). Values are expressed as Mean ± SEM (*n* = 3 replicates). * *p*-value ≤ 0.05 compared to the respective controls. ns—non-significant. Magnification 10X, scale 200 µm.

EtOH exposure to AT2 cells significantly reduced the levels of DPPC (Figure 13A) and expression of SP-C (Figure 13B) at all time points studied. The DPPC levels were comparable at 6, 24 and 72 h. However, the SP-C fluorescence intensity was markedly reduced in EtOH-treated cells at 72 h than at 24 and 6 h. Thus, extended EtOH exposure to AT2 cells caused a significant reduction of SP-C expression, which could be relevant to the subjects diagnosed with AUD or chronic alcohol use/abuse experiencing various lung-related complications.

### 5.3. AMPKα/ER Stress Signaling and Oxidative Stress

**Figure 14:**
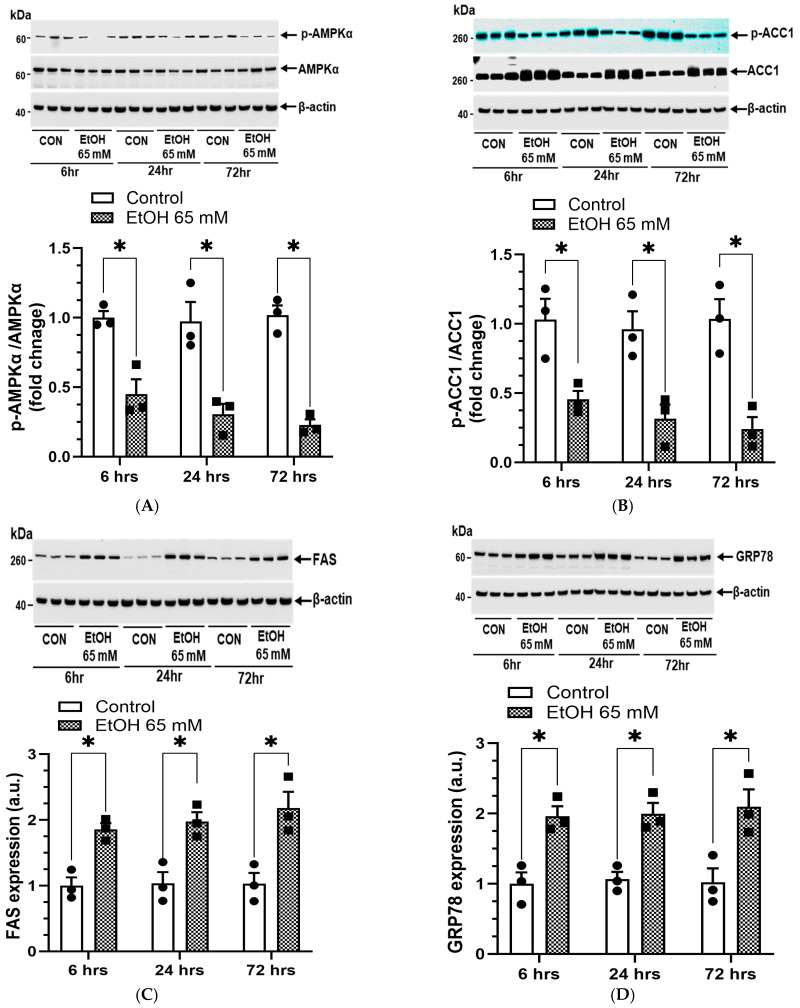

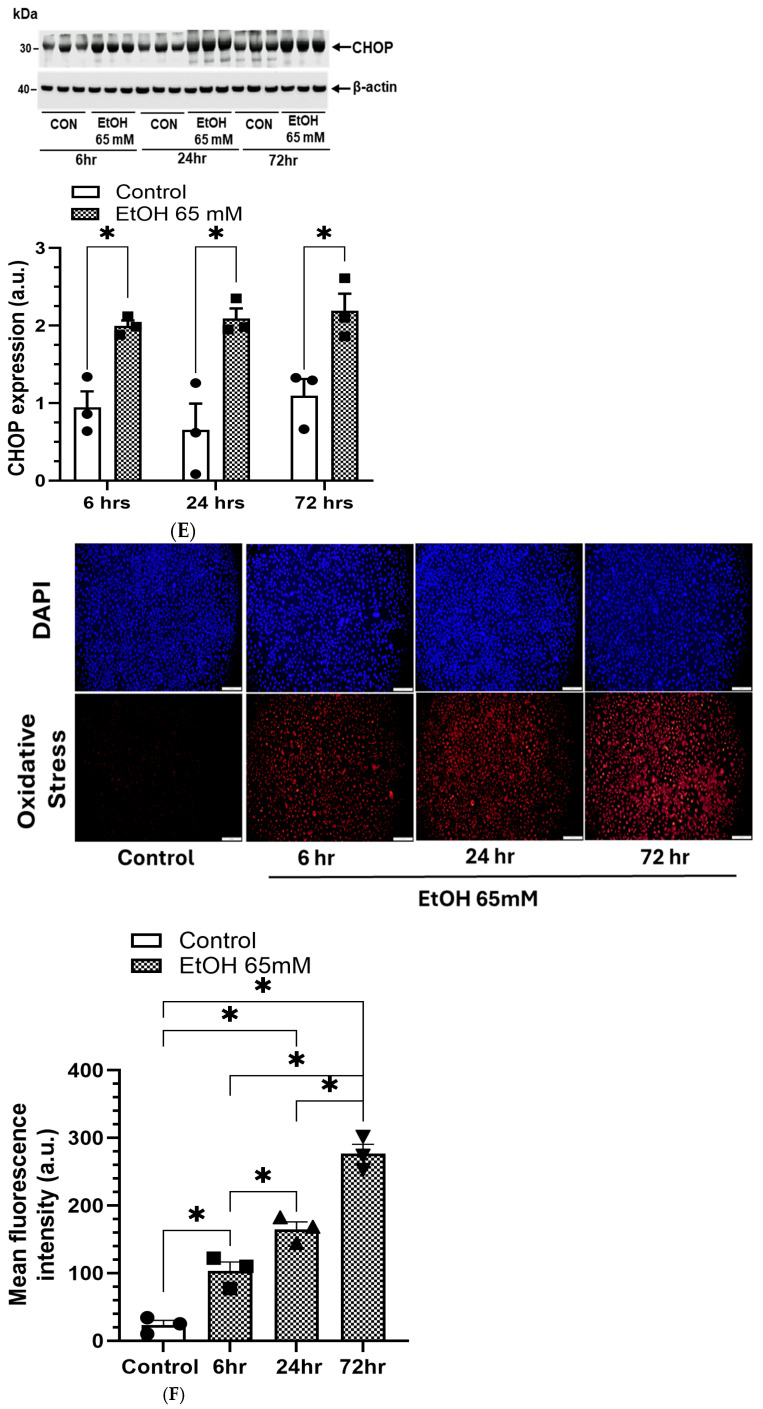
Time-dependent changes in the AMPKα and ER stress signaling, and oxidative stress in AT2 cells treated with 65 mM EtOH for 6, 24, and 72 h. Representative immunoblots, and corresponding bar graphs show protein expression for p-AMPKα/AMPKα (**A**), p-ACC1/ACC1 (**B**), FAS (**C**), GRP78 (**D**), and CHOP (**E**). Intensities are normalized to β-actin (loading control). Oxidative stress (**F**), DAPI -upper panel, CellROX orange dye fluorescence-lower panel. Quantification of fluorescence intensity measured using ImageJ. Values are expressed as Mean ± SEM (*n* = 3 replicates). * *p*-value ≤ 0.05 with respect to the controls. Magnification 10X, scale 200 µm.

AMPKα inactivation and ER/Oxidative stress increased time-dependently in EtOH-treated AT2 cells. The maximum inactivation of AMPKα (Figure 14A) with decreased p-ACC1 (Figure 14B), and increased expression of ACC1 (Figure 14B) and FAS (Figure 14C) involved in lipogenesis, was found at 72 h. Furthermore, elevated levels of GRP78 and CHOP (markers for ER stress) were comparable at all three time points (Figure 14D,E). AT2 cells treated with EtOH showed significant oxidative stress at all time-points studied herein, the highest being at 72 h (Figure 14F). Thus, a time-dependent sustained inactivation of AMPKα, ER/oxidative stress, and several-fold increased formation of FAEEs in AT2 cells treated with EtOH, as found in this study, could be key factors contributing to the pathogenesis of ARLD.

## 6. Discussion

EtOH exposure exerts multifaceted cytotoxic effects on immortalized human AT2 cells by disrupting cellular homeostasis at multiple levels, including inflammation, redox balance, mitochondrial bioenergetics, lipid metabolism, and surfactant production. Most of the effects were both concentration and time-dependent, to be mediated via non-oxidative EtOH metabolism to FAEEs, dysregulation of AMPKα signaling, ER/oxidative stress, and mitochondrial stress. These molecular changes closely reflect the pathological features of ARLD’ characterized by epithelial barrier dysfunction, impaired surfactant production, defective host defense, and increased susceptibility to pulmonary infections [2,3,4,9].

The EtOH concentrations (22, 65, and 130 mM) used in this study correspond to blood alcohol concentrations (BACs) of approximately 0.1, 0.3, and 0.6%, respectively, commonly observed in chronic alcoholics [41]. While direct measurement of EtOH levels in the human alveolar epithelial lining fluid are limited, studies have demonstrated that EtOH equilibrates rapidly across the alveolar–capillary barrier, resulting in tissue concentrations comparable to those found in systemic circulation [3,51]. This equilibrium is further supported by exhaled breath EtOH data and diffusion modeling studies, which collectively indicate that the alveolar microenvironment experiences EtOH exposure proportional to circulating levels [52,53]. Therefore, the EtOH concentrations used in this study reflect physiologically achievable conditions that simulate the alveolar epithelial microenvironment and represent exposure levels likely to occur in the alveolar milieu of individuals with chronic alcohol use and AUD. In addition, these concentrations are consistent with our prior in vitro studies examining EtOH-induced cytotoxicity and metabolic dysregulation in pulmonary cell systems [28,34,35], thereby enhancing the physiological and translational relevance of the present findings. By using EtOH concentrations that mimic blood alcohol levels typically observed in chronic alcohol users or AUD, our findings provide mechanistic insight into how sustained EtOH exposure compromises alveolar epithelial function and contributes to the pathogenesis of ARLD.

Although EtOH did not significantly reduce overall cell viability, it caused substantial LDH release into the extracellular medium, indicating a loss in membrane integrity and subsequent membrane damage. This apparent disconnect between viability and LDH release suggests that EtOH and/or its metabolism may compromise membrane integrity before the onset of overt metabolic failure and cell death, consistent with previous studies indicating that EtOH-induced lipid bilayer fluidity and tight junction stability in pulmonary epithelial cells are compromised [54,55]. The esterification of endogenous fatty acids by EtOH to form lipophilic, non-oxidative metabolites (FAEEs), particularly at high concentrations, explains altered cell membrane integrity and fluidity and LDH release.

EtOH exposure also significantly augmented the secretion of proinflammatory cytokines IL-6, TNF-α, and MCP-1, which are similarly elevated in bronchoalveolar lavage fluid from individuals with AUD and are linked to alveolar macrophage dysfunction and impaired pulmonary immunity [3,11,28,56]. The induction of these cytokines and chemokines in AT2 cells underscores their active role in initiating local inflammatory responses within the alveolar microenvironment and compromising the pulmonary barrier defense mechanism(s).

Mitochondria are central to cellular energetics and redox homeostasis in the alveolar epithelium, and their dysfunction likely contribute to the pathogenesis of ARLD [57,58]. The significant induction of CYP2E1 following EtOH exposure in AT2 cells can generate reactive oxygen species (ROS), leading to lipid peroxidation, protein oxidation, and DNA damage resulting in oxidative stress [59,60]. Additionally, EtOH induced pool of free fatty acids, and their oxidation can also cause oxidative stress. However, both possibilities need to be established.

Our observation of reduced mitochondrial respiration, including basal, maximal, and ATP-linked oxygen consumption rates, alongside diminished spare respiratory capacity and coupling efficiency, suggests that mitochondrial dysfunction is a major contributor to this oxidative stress and eventual cell injury as compared to CYP2E1-induced EtOH metabolism. These findings corroborate well with prior reports that EtOH exposure impairs mitochondrial function in pulmonary epithelial and endothelial cells by altering mitochondrial membrane potential and inducing the transition of mitochondrial permeability [35,61,62,63]. The observed decline in total, mitochondrial, and glycolytic ATP production reflects a failure of global energy or its functional impairment. Therefore, we hypothesize that EtOH-induced suppression of both oxidative phosphorylation and glycolysis could be associated with its non-oxidative metabolism to FAEEs. Furthermore, the disruption of both oxidative phosphorylation and glycolytic ATP synthesis, is likely through suppression of metabolic enzymes regulating ATP production pathways. Although enzyme-specific analyses were not performed in this study, a reduced ATP generation is consistent with potential inhibition of ATP synthase, pyruvate dehydrogenase (PDH), and phosphofructokinase (PFK), as reported in other EtOH models [64]. Future studies evaluating the expression and activity of these enzymes should define the mechanistic basis of EtOH-induced bioenergetic failure in AT2 cells.

EtOH exposure also induces marked mitochondrial stress in AT2 cells, compromising cellular bioenergetics and causing oxidative stress. Mitochondrial homeostasis relies on the coordinated regulation of biogenesis, fission, and fusion, mediated by PGC-1α, DRP1, and MFN2, respectively [65]. Disruption of these pathways promotes mitochondrial fragmentation, ROS overproduction, and loss of respiratory function [65,66]. Previous studies demonstrate that EtOH exposure impairs mitochondrial respiration and disrupts mitochondrial function, leading to oxidative injury and alveolar epithelial dysfunction [63,67,68,69]. Together our findings within this regulatory network provide mechanistic insight into how mitochondrial stress contributes to surfactant protein impairment and alveolar toxicity.

The energy-sensing kinase AMPKα plays a central role in maintaining cellular energy balance, particularly under stress. EtOH exposure resulted in marked deactivation of AMPKα and its upstream regulator LKB1, with no significant involvement of CaMKKβ. This suggests that alcohol selectively impairs the LKB1–AMPKα axis in AT2 cells. This is in line with previous reports that alcohol impairs AMPKα signaling, leading to metabolic imbalance and lipotoxicity [28,38,47].

Consistent with AMPKα inactivation, an increased expression of lipogenic proteins (ACC1 and FAS), decreased p-ACC1 and CPT1A, an enzyme involved in β-oxidation of fats, are indicative of a lipogenic shift and impaired lipid catabolism in the pathogenesis of ARLD. Such metabolic reprogramming may contribute to intracellular lipid accumulation and enhanced formation of FAEEs, lipotoxicity, mitochondrial toxicity, and oxidative stress, contributing to the lung injury [70,71].

Importantly, an increased expression of EtOH-metabolizing enzymes, CYP2E1 and CEL, contrary to ADH1, suggests a limited capacity of AT2 cells to metabolize EtOH oxidatively via classical hepatic ADH1 pathways in the lungs. Instead, the non-oxidative pathway, mediated by CEL, appears to be a dominant route in AT2 cells to form FAEEs accumulating in the lungs of alcoholic subjects.

Of importance, this could be the first report to demonstrate that human AT2 cells efficiently metabolize EtOH to FAEEs via esterification of endogenous fatty acids. These lipophilic esters integrate into the mosaic of cellular membranes, disrupt membrane fluidity, and impair organelle function, inducing cytotoxicity and mitochondrial stress [21,24]. FAEEs have been shown to accumulate in the lungs to cause cytotoxicity to pulmonary endothelial and epithelial cells via mitochondrial depolarization [3,25,27,35]. In addition, significant mitochondrial stress along with decreased mitochondrial ATP production rate, and inhibition of CPT1A in AT2 cells treated with EtOH, further suggests that FAEEs are the major EtOH metabolites inducing mitochondrial dysfunction, oxidative stress, and affect overall cellular health [22]. Possibly, once formed, FAEEs are transported to mitochondria by the same trafficking proteins involved in the transport of fatty acids.

While our data shows substantial FAEE accumulation in EtOH-treated AT2 cells, these findings are correlative and do not definitively establish FAEEs as the direct mediators of cytotoxicity. Our prior studies in primary human bronchial epithelial cells and alveolar macrophages demonstrated that FAEEs elicit stronger cytotoxic and inflammatory responses than EtOH itself [28,35], supporting their pathogenic potential. Future studies will employ purified FAEE species and target inhibition of FAEE synthesis to determine their causal role in EtOH-induced AT2 cell injury and mitochondrial dysfunction.

The metabolic shift toward non-oxidative pathways in AT2 cells treated by EtOH is further supported by an increased level of FAEEs following ADH1 inhibition with 4-methylpyrazole. These findings are significant because they suggest that an impaired classical hepatic oxidative metabolism of alcohol by ADH1 as observed in individuals with AUD [16,17,18,20], could exacerbate cellular injury via the formation of FAEEs. Our findings strongly suggest that nonoxidative metabolism of EtOH represents a critical yet underrecognized contributor to EtOH-induced lung alveolar epithelial cell injury. Further studies are needed to establish the role of individual FAEEs in the pathogenesis of ARLD.

Surfactant dysregulation can impair alveolar stability, reduce lung compliance, and exacerbate inflammation, contributing to the clinical manifestations of ARLD [2,4,72]. Significantly decreased expression of surfactant proteins, SP-B and SP-C, as well as the levels of DPPC, the principal phospholipid component of pulmonary surfactants, strongly suggest that EtOH exposure increases alveolar surface tension, resulting in the collapse of alveoli or a compromised state of its vital function. Therefore, measuring the real-time surface tension in AT2 cells treated with EtOH and/or FAEEs could provide a metabolic basis for the ARLD phenotype. Given the central role of surfactants in reducing alveolar surface tension during breathing, maintaining lung compliance is critical to prevent atelectasis. Reduced DPPC levels could also have profound consequences disrupting the alveolar stability and gas exchange processes. Therefore, our findings also help explain the increased incidence of respiratory failure and ARDS in individuals with AUD, as previously described in clinical studies [73,74].

Previous studies have implicated oxidative and ER stress in the downregulation of surfactant biosynthesis [75,76,77,78]. Therefore, an increased expression of ER stress markers GRP78 and CHOP following EtOH exposure further links ER dysfunction to impaired surfactant production. Interestingly, we did not observe significant activation of XBP1 splicing, suggesting an incomplete or dysregulated UPR. Unresolved ER stress alone can lead to apoptosis, inflammation, and impairment of surfactant protein folding and trafficking, further exacerbating lung injury [77,79].

In addition, the concomitant deactivation of AMPKα and uniform reduction in SP-B and SP-C expression further indicate that EtOH-induced loss of surfactant proteins results from transcriptional repression rather than selective degradation. This interpretation aligns with prior studies that oxidative and ER stress inhibit the transcription factors TTF-1 (NKX2-1) and FoxA2, leading to reduced SP-B/SP-C gene expression [80,81,82]. Persistent ER stress and impaired transcriptional regulation may therefore converge to disrupt surfactant homeostasis, promoting alveolar instability and progression of ARLD. Furthermore, prolonged exposure to EtOH progressively worsened cellular injury, which included increased formation of FAEEs, greater AMPKα inactivation, reduced surfactants, and enhanced ER/oxidative stress at 72 h. These findings suggest that EtOH exposure, if prolonged or repeated, could result in a greater chronic dysfunction in the alveolar epithelium.

Our findings highlight the nonoxidative metabolism of EtOH to FAEEs as the key mediator of alveolar epithelial dysfunction. Beyond these direct metabolic and molecular effects in AT2 cells, chronic alcohol exposure may also influence pulmonary injury through systemic pathways involving inter-organ and neuroimmune communication. Chronic alcohol exposure induces systemic alterations across the gut–lung–brain axis, linking metabolic, immune, and neurochemical dysfunctions. EtOH disrupts neurotransmitter signaling and gut barrier integrity, promoting dysbiosis and microbial translocation that drive systemic inflammation and exacerbate lung injury [83,84]. These findings underscore the interplay between neuroimmune and peripheral pathways in ARLD. Thus, future studies incorporating neuroendocrine and microbiome perspectives can also define systemic mechanisms of alcohol-induced pulmonary injury.

Our findings identify enhanced FAEE formation and dysregulated AMPKα signaling as likely central mechanisms of EtOH-induced AT2 cell injury. Pharmacologic AMPKα activation has been shown to restore mitochondrial function and reduce inflammation in experimental lung injury models. Agents such as AICAR, A-769662, and metformin improve p-AMPKα signaling and attenuate oxidative and inflammatory responses, supporting AMPKα as a promising therapeutic target [35,85]. In parallel, inhibition of FAEE synthesis through inhibition of CEL activity reduces FAEE accumulation and prevents mitochondrial dysfunction in EtOH-related injury models [86]. Future studies should determine whether AMPKα activation or FAEE inhibition can mitigate EtOH-induced cytotoxicity and restore surfactant homeostasis in AT2 cells. The noticeable limitations of this study include the possibility that in vitro EtOH exposure in AT2 cells alone may not capture the full spectrum of immune, mechanical, and systemic interactions present in the alveolar microenvironment during chronic alcohol use, which could be orchestrated by a variety of pulmonary cells. Therefore, employing a co-culture system with alveolar macrophages and/or fibroblasts may provide a physiologically relevant model to investigate intercellular signaling and the progression of alcohol-induced lung injury. Future studies, by incorporating whole AT2 cell lipidomics, could define the surfactant alteration patterns over time and identify the target surfactant(s).

While the immortalized human AT2 cell line used in this study offers valuable and reproducible findings to delineate the direct metabolic, molecular, and mitochondrial responses to EtOH exposure, it does not fully recapitulate the physiological complexity of the alveolar niche. These cells preserve key phenotypic features of native AT2 cells—such as surfactant protein expression and lipid metabolic activity—but lack interactions with other pulmonary cell types, including macrophages, fibroblasts, and endothelial cells, which modulate immune signaling and barrier function. Moreover, immortalization may alter differentiation capacity and stress signaling pathways, potentially influencing the magnitude of EtOH-induced responses. Therefore, future studies using primary human AT2 cells, air–liquid interface cultures, or organotypic co-culture and lung-on-chip systems could validate these findings under conditions that more closely mimic the in vivo alveolar environment.

## 7. Conclusions

This study provides a comprehensive mechanistic insight into how EtOH exposure causes injury to AT2 cells. We demonstrated that EtOH, particularly at concentrations ≥ 65 mM (a clinically relevant concentration observed in the blood of chronic alcoholics), induces a multifactorial injury response that includes cytotoxicity, oxidative and ER stress, mitochondrial dysfunction, and dysregulation of cellular energetics and lipid metabolism. A critical finding is the identification of an EtOH non-oxidative metabolic pathway, catalyzed by CEL, resulting in the formation of FAEEs that are likely to contribute to cellular injury and mitochondrial dysfunction.

Importantly, EtOH exposure inactivates the LKB1–AMPKα signaling axis, promotes lipogenesis, suppresses fatty acid β-oxidation, and impairs ATP generation. These metabolic alterations coincide with reduced levels of DPPC and surfactant proteins SP-B and SP-C, indicating profound disruption of surfactant homeostasis—a hallmark of AT2 cell dysfunction. The observed inflammatory cytokine release further highlights the potential of EtOH to prime the alveolar environment for heightened injury in response to secondary insults.

Altogether, our findings demonstrate that EtOH exposure impairs AT2 cell function through multiple interrelated mechanisms, which may underline the increased susceptibility to lung injury, ARDS, and impaired alveolar repair observed in individuals with AUD. Therefore, targeting alcohol metabolism and AMPKα signaling should enable us to identify molecular targets and develop novel therapeutic strategies for ARLD.

## Figures and Tables

**Table 1:** Primer sequences used for qPCR analysis.

Gene	Forward Primer (5′–3′)	Reverse Primer (5′-3′)
*Cel*	TCGTGGAAGGCGTCAATAAG	CAGGATGTGGCTGAGGATTT
*Cyp2e1*	CTCGTGCTCTTGCCCTATTT	TGCTGACGGCGTTCTTTATC
*SfptA1*	GGCTATTGACTGAGCACCTATC	GGTCCCGTGACATTGTGTAA
*SfptA2*	GAGAGATGGTGTCAAAGGAGAC	TCCAGGCAGCCCATTATTC
*SfptB*	TACTCACTGGGATGAGGTTAGG	GGACACTTCCAGGCATTTCA
*SfptC*	GCACCTGAAACGCCTTCTTATC	TGCTCATCTCCAGAACCATCTC
*SfptD*	GGCTACCTGGAAGCAGAAAT	CTCCACTGAGCTACACATGAC
*Rplpo*	GGAGAAACTGCTGCCTCATATC	CAGCAGCTGGCACCTTATT

## Data Availability

The raw data supporting the conclusions of this article will be made available by the authors on request.

## Abbreviations

The following abbreviations are used in this manuscript:

ACC1	Acetyl CoA carboxylase 1
ADH1	Alcohol dehydrogenase 1
AMPKα	AMP activated protein kinase α
ARLD	Alcohol-related lung disease
AT2	Alveolar type 2 epithelial cells
ATP	Adenosine triphosphate
AUD	Alcohol use disorder
BAC	Blood alcohol concentration
CEL	Carboxyl ester lipase
CPT1A	Carnitine palmitoyltransferase 1A
CYP2E1	Cytochrome P450 2E1
DPPC	Dipalmitoylphosphatidylcholine
ECAR	Extracellular acidification rate
EIF2α	Eukaryotic translation initiation factor 2α
EtOH	Ethanol
ER	Endoplasmic reticulum
FAEEs	Fatty acid ethyl esters
GROα	Growth-Regulated Oncogene
GRP78	Glucose-regulated protein 78
IRE1α	Inositol-requiring enzyme 1α
LDH	Lactate dehydrogenase
LKB1	Liver kinase B1
OCR	Oxygen consumption rate
PERK	Protein kinase RNA-like ER kinase
RANTES	Regulated upon activation, normal T-cell expressed and secreted
SP-C	Surfactant protein C
XBP1	X-Box binding protein 1
UPR	Unfolded protein response
